# Assessment of Ankaferd Blood Stopper in experimental liver ischemia reperfusion injury

**DOI:** 10.3906/sag-2004-240

**Published:** 2020-08-26

**Authors:** Abdullah DURHAN, Koray KOŞMAZ, Marlen SÜLEYMAN, Mesut TEZ, Abdullah ŞENLİKCİ, Can ERSAK, Yılmaz ÜNAL, Recep PEKÇİCİ, Fatih KARAAHMET, Mehmet ŞENES, İlknur ALKAN KUŞABBİ, Eylem Pınar ESER, Sema HÜCÜMENOĞLU

**Affiliations:** 1 Department of Surgical Oncology, Department of General Surgery, Ministry of Health Ankara Training and Research Hospital, Ankara Turkey; 2 Department of Gastrointestinal Surgery, Department of General Surgery, Ministry of Health Ankara Training and Research Hospital, Ankara Turkey; 3 Department of General Surgery, Ministry of Health Ankara Training and Research Hospital, Ankara Turkey; 4 Department of Gastroenterology and Hepatology, Department of Internal Medicine, Ministry of Health Ankara Training and Research Hospital, Ankara Turkey; 5 Department of Biochemistry, Ministry of Health Ankara Training and Research Hospital, Ankara Turkey; 6 Department of Pathology, Ministry of Health Ankara Training and Research Hospital, Ankara Turkey

**Keywords:** Ischemia reperfusion injury, Ankaferd Blood Stopper, liver

## Abstract

**Background/aim:**

To investigate possible protective effects of Ankaferd Blood Stopper® (ABS) in an experimental liver ischemia reperfusion injury (IRI) model.

**Materials and methods:**

The study was carried out on 30 female rats separated into 3 groups as sham, control (IRI), and treatment (IRI + ABS) groups. In the IRI + ABS group, 0.5 mL/day ABS was given for 7 days before surgery. In the IRI and IRI + ABS groups, the hepatic pedicle was clamped for 30 min to apply ischemia. Then, after opening the clamp, 90-min reperfusion of the liver was provided. Blood and liver tissue samples were taken for biochemical and histopathological analyses.

**Results:**

Compared to the sham group, the IRI group had significantly higher levels of alanine aminotransferase (ALT), aspartate aminotransferase (AST), total oxidant status (TOS), malondialdehyde (MDA), fluorescent oxidant products (FOP) and lower expression of albumin and total antioxidant status (TAS) (P < 0.05). Compared to the IRI group, the IRI+ABS group showed lower expression of AST, ALT, TOS, MDA and FOP and higher expression of albumin and TAS (P < 0.05). In the histopathological analysis, congestion scores were statistically significantly lower in the IRI + ABS group than in the IRI group.

**Conclusions:**

ABS has a strong hepatoprotective effect due to its antioxidant and antiinflammatory effects and could therefore be used as a potential therapeutic agent for IRI.

## 1. Introduction

Ischemia reperfusion injury (IRI) is a critical condition which presents serious therapeutic challenges for clinicians. It occurs due to the restoration of blood supply following prolonged ischemia. During the ischemic period, anaerobic metabolism and dysfunction of the electron transport chain in mitochondria are promoted. Cell swelling and disrupted enzyme activity in the cytoplasm occurs as a result of decreased adenosine triphosphate (ATP) production because of dysfunction of the ion exchange channels. After blood flow restoration (reperfusion), oxidative stress, reactive oxygen species, and local inflammation leading to secondary injury are induced by electrolyte imbalance and mitochondrial damage. Finally, the retention of reactive oxygen species leads to cell death by means of autophagy, mitoptosis, necrosis, necroptosis, and apoptosis [1–4].

IRI can affect multiple organs including the liver, heart, intestine, kidneys, and brain and is also related to grave clinical manifestations such as systemic inflammatory response syndrome, acute heart failure, myocardial hibernation, cerebral dysfunction, multiple organ dysfunction syndrome, and liver dysfunction. In hepatic IRI pathogenesis, several factors and pathways play a role, such as mitochondria, anaerobic metabolism, intracellular calcium overload, oxidative stress, cytokines and chemokines, and liver Kupffer cells and neutrophils [5]. It is possible for hepatic IRI to cause serious liver dysfunction. It may even inflict irreversible damage in the body, which then results in a series of multiple organ dysfunction [5,6]. Hepatic IRI occurs during different surgical interventions implemented for intrahepatic lesions or liver transplantation. These surgical procedures need a certain period of ischemia to allow total vascular exclusion or Pringle’s manoeuvre (inflow control) [7]. Although several studies have investigated nitric oxide, hesperidin, diosmin, infliximab, calcium dobesilate and glycyrrhetinic acid, to prevent or decrease hepatic IRI, there is still no agent commonly used in clinical practice [5, 8–10].

Ankaferd Blood Stopper® (ABS) is a most well known topical hemostatic agent and contains standardized plant extracts taken from Glycyrrhiza glabra, Urtica dioica, Vitis vinifera, Thymus vulgaris, and Alpinia officinarum [11]. The herbs listed here have effects on blood cells, endothelium, the proliferation of cells, angiogenesis, and vascular dynamics, and vascular mediators. Moreover, some also have antioxidant and antiinflammatory effects [12,13], which may be useful for hepatic IRI. Only a limited number of studies have been carried out to investigate how ABS affects liver surgery and hepatic IRI. The aim of this study was to investigate the possible protective effects of ABS in an experimental liver IRI model. 

## 2. Materials and methods

Approval for the study was granted by the Animal Ethics Committee. The experimental procedures and technique of this study met the requirements of the national guidelines for the use and care of laboratory animals.

### 2.1. Animals and experimental surgical procedure

The study sample consisted of 30 female adults Wistar albino rats, each with a weight of 230 ± 22 gr. The rats were housed in cages made of wire netting at a permanent temperature of 21 ± 2 °C with a 12-h light/dark cycle. The animals had a diet consisting of standard laboratory supply chow and water ad libitum. Access to food was stopped 12 h before anesthesia and to water 2 h prior to anesthesia for the rats. The same team conducted the anesthesia and surgical intervention stages under sterile conditions. Before the intervention procedures, anaesthesia was administered of 50 mg/kg ketamine hydrochloride (Ketalar; Parke-Davis, Detroit, MI, USA) and 5mg/kg Xylazine (Rompun; Bayer AG, Leverkusen, Germany) via intramuscular injection.

The rats were randomly assigned to 3 groups of 10 rats: the sham group, the control group, and the treatment group. 

In Group 1 (sham), only hepatic pedicle was induced. 

In Group 2 (control) (IRI), ischemia was performed by clamping the hepatic pedicle for 30 min. Following the ischemia application, the clamp was opened and 90-min reperfusion of the liver was performed.

In Group 3 (treatment) (IRI + ABS), 0.5 mL/day ABS was administered via an orogastric tube for 7 days before the surgery. Ischemia was applied by clamping the hepatic pedicle for 30 min, then the clamp was opened to provide reperfusion for 90 min. 

After the completion of these procedures, all the rats were euthanized with ketamine overdose. After the laparotomy, blood samples were taken from the aorta and tissue samples were obtained from liver for histopathological and biochemical analysis. 

### 2.2. Histopathological analysis

The rat liver tissue samples were fixed in a solution containing 10% formaldehyde for a period of 24 h and then embedded in blocks of paraffin. From these, sections 4 µm in thickness were cut with a microtome (Leica RM 2155 RT, Leica Microsystems GmbH, Wetzlar, Germany). Hematoxylin and eosin were used to stain these tissue sections and then the stained sections were analyzed. The samples were examined histopathologically using an Olympus BX51TF light microscope (Olympus Corporation, Tokyo, Japan) by a pathologist who was blinded to the groups. In the histopathological evaluation, the modified Suzuki scoring system [14] from 0 to 4 was used to grade sinusoidal congestion, liver cell vacuolization and hepatocyte necrosis. The modified Suzuki scoring system is shown in Table 1. 

**Table 1:** The histopathological scoring system (Suzuki) of the liver samples [14].

Grade	Sinusoidal congestion	Vacuolization	Necrosis
0	None	None	None
1	Mild	Mild	Simple cell necrosis
2	Moderate	Moderate	0%–30 %
3	Severe	Severe	30%–60 %
4	Excessive	Excessive	>60%

### 2.3. Biochemical analysis

Liver functions in the serum and oxidative stress parameters were evaluated. The measurement of aspartate aminotransferase (AST), alanine aminotransferase (ALT), albumin, total and direct bilirubin was performed using a Roche Cobas 8000 chemistry analyzer (Roche Diagnostics, Risch-Rotkreuz, Switzerland).

The liver tissue samples were kept at –80 °C until the day of the laboratory and histological assessment. Total oxidant status (TOS), total antioxidant atatus (TAS), malondialdehyde (MDA) and fluorescent oxidation products (FOP) levels were determined. The levels of TOS and TAS were measured by applying the TOS and TAS kits of Rel Assay Diagnostic to the Roche Cobas 6000 instrument. 

In the measurement of TAS, the antioxidants in the sample reduce the radical 2,2′-Azino-bis(3-ethylbenzothiazoline-6-sulfonic acid) (ABTS), which is dark blue-green colored to a colorless reduced ABTS form. Absorbance change at 660 nm is associated with the level of total antioxidants present in the sample. The calibration of the assay was carried out using a stable antioxidant standard solution of Trolox equivalent, an analogue of vitamin E.

In the measurement of TOS, ferrous ion-chelator complex is oxidized to ferric ion via to oxidants within the sample. Enhancer molecules, which can be found in great numbers in the medium of reaction, prolong the reaction of oxidation. Ferric ion produces a complex colored with chromogen in an acidic medium. The intensity of color, which can be spectrophotometrically measured, is associated with the total quantity of oxidants present within the sample. Hydrogen peroxide was used to calibrate the assay. The results of TAS and TOS were stated as µmol/g protein.

MDA functions as an indicator of lipid peroxidation and is known to be an index of tissue injury. The fluorometric method was used to measure MDA levels as described by Wasowicz et al. [15]. Following the reaction between thiobarbituric acid (TBA) and MDA, the product of the reaction was isolated in butanol. Then spectrofluorometric measurement was taken at a wavelength of 547 nm for emission and 525 nm for excitation. The standard was designated as a solution of 0–5 µmol/L 1,1’,3,3’ tetraethoxypropane. In order to measure the MDA levels in the tissue, 50 µL of homogenate was added into glass tubes of 10 mL, each of which contained 1 mL of distilled water. Following this step, a 1-mL solution that contained 29 mmol/L TBA was added to acetic acid and mixed. Then, the samples were exposed to a temperature of 95–100°C for 1 h using a water bath. After cooling the heated samples, they were mixed with 25 µL of 5 mol/L hydrochloric acid (HCL) and agitation was applied for 5 min to extract the mixture of the reaction using 3.5 mL n-butanol. After the butanol phase was separated through centrifugation for 10 min at 1500 g, a fluorometer (HITACHI F-2500) was used to measure the fluorescence in the butanol extract at 547 nm and 525 nm wavelengths for emission and excitation, respectively. Solutions of 0–5 µmol/L 1,1’,3,3’ tetraethoxypropane were utilized as the standard. The levels of MDA were shown as nmol/g protein. Tissues that were homogenized were isolated by means of ethanol-ether (3/1, v/v) for the measurements of FOP. Following this, a spectrofluorometer was used to evaluate these tissues at wavelengths of 360 and 430 (wavelengths for excitation/emission) [16]. 

### 2.4. Statistical analysis

The data were analyzed statistically with SPSS 23.0 software (IBM Corp., Armonk, NY, USA). The Shapiro–Wilk’s test, skewness and kurtosis values were used to analyze the data distribution. All data were expressed as mean ± standard deviation (SD). The statistical analysis of the results was performed using unpaired Student’s t-tests and ANOVA models (with Tamhane’s post hoc test) with normally distributed data. For other type of data, the Mann–Whitney U test and the Kruskal–Wallis tests were used. The association between categorical variables was tested using chi-square test or Fisher’s exact test. Significance was considered when P < 0.05 and supported by Bonferroni multiple testing correction. 

## 3. Results

### 3.1. Histopathological findings

The histopathological results of the study are shown in Table 2. The lowest congestion scores were found in the sham group and the highest congestion scores in the IRI group. Congestion scores were determined to be statistically significantly lower in the IRI + ABS group than in the IRI group (P = 0.005). 

**Table 2:** The mean histopathological scores of the study groups.

Groups	Congestion (mean ± SD)	Vacuolisation (mean ± SD)	Necrosis (mean ±SD)
Group 1 (sham)	0.60 ± 0.51^x^	0.00 ± 0.00z	0.00 ± 0.00
Group 2 (IRI)	2.20 ± 0.78^x.y^	0.80 ± 0.78^z^	0.30 ± 0.28
Group 3 (IRI+ABS)	1.0 ± 0.66^y^	0.20 ± 0.42	0.10 ± 0.10

Significantly different. ^x^P < 0.001 for group 1 versus group 2, ^y^P =0.005 for group 2 versus group 3, ^z^P = 0.03 for group 1 and group 2. IRI: Ischemia reperfusion injury, ABS: Ankaferd Blood Stopper.

In the histopathological assessment, vacuolization and necrosis were not seen in the sham group and were detected more in the IRI group compared to the IRI + ABS group. There was statistical significance between the sham and IRI groups in respect of vacuolization (P = 0.03), and no statistically signiﬁcant difference was observed between the IRI and IRI+ABS groups (P = 0.15). The groups displayed no statistical significance in respect of the necrosis scores (group sham vs. IRI: P = 0.22, IRI vs. IRI + ABS: P = 0.64) (Figure). 

**Figure:**
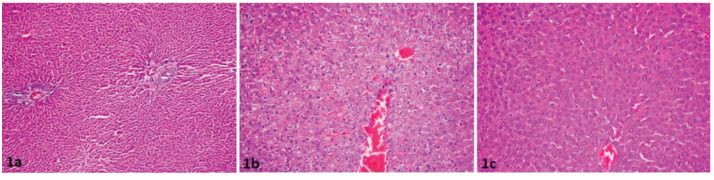
Histopathological findings are shown in figure. There was not any congestion and vacuolization in sham group (Figure 1a). While moderate congestion and vacuolization were seen in control group (Figure 1b), mild congestion and vacuolization were seen in treatment group (Figure 1c).

### 3.2. Biochemical findings- liver function test

Compared to the IRI and IRI+ABS groups, the sham group had the lowest ALT and AST values and the highest albumin values. The analysis revealed a difference of statistical significance (P < 0.05). The IRI+ABS group was determined to have higher albumin levels and lower AST and ALT levels than the IRI group (P < 0.05).

There was no statistical significance between the groups in respect of total bilirubin and direct bilirubin values (P > 0.05). The test results of the liver function tests are shown in detail in Table 3. 

**Table 3:** Liver function parameters of groups.

Groups	ALT (U/L) (mean ± SD)	AST (U/L) (mean ± SD)	Albumine (g/dL) (mean ± SD)	T Bilirubin (mg/dL) (mean ± SD)	D Bilirubin (mg/dL) (mean ± SD)
Group 1(Sham)	66.5 ± 11.36^xy^	176.6 ± 29.44^xy^	3.80 ± 0.15^xp^	0.06 ± 0.01	0.03 ± 0.01
Group 2(IRI)	271.40 ± 85.9^xz^	566.10 ± 66.88^xt^	3.20 ± 0.22^xu^	0.04 ± 0.01	0.02 ± 0.00
Group 3(IRI+ABS)	139.1 ± 31.75^yz^	322.80 ± 60.65^yt^	3.52 ± 0.12^pu^	0.07 ± 0.01	0.03 ± 0.01

Significantly different. ^x^P < 0.001 for group 1 versus group 2, ^y^P < 0.001 for group 1 versus group 3, ^z^P = 0.002 for group 2 and group 3, ^t^P < 0.001 for group 2 and group 3, ^p^P = 0.001 for group 1 versus group 3, ^u^P = 0.005 for group 2 versus group 3. AST: Aminotransferase, ALT: Alanine aminotransferase, SD: Standard deviation, T: Total, D: Direct.

### 3.3. Biochemical findings- oxidative stress parameters 

 The oxidative stress parameter values are shown in detail in Table 4. 

**Table 4:** Oxidative stress parameters of the groups.

Groups	TAS (mean ± SD) (µmol/g protein)	TOS (mean ± SD) (µmol/g protein)	MDA (mean ± SD) (nmol/g protein)	FOP (mean ± SD) (FP/g protein)
Group 1(Sham)	204.43 ± 13.27^xy^	17.6 ± 3.99^x^	9.17 ± 3.12^xp^	6.23 ± 1.4^xp^
Group 2(IRI)	130.82 ± 20.41^xz^	33.44 ± 8.36^xt^	26.64 ± 6.07^xz^	16.76 ± 2.52^xu^
Group 3(IRI+ABS)	174.45 ± 21.14^yz^	20.55 ± 3.13^t^	15.5 ± 3.5^pz^	10.34 ± 2.23^pu^

Significantly different, ^x^P < 0.001 for group 1 versus group 2, ^y^P = 0.005 for group 1 versus group 3, ^z^P = 0.001 for group 2 and group 3, ^t^P = 0.002 for group 2 and group 3, ^p^P = 0.001 for group 1 versus group 3, ^u^P = 0.005 for group 2 versus group 3, ^xu^ P < 0.001 for group 2 versus group 3. TAS: Total antioxidant status, TOS: Total oxidant status, MDA: Malondialdehyde, FOP: Fluorescent oxidant products.

The values of TOS, FOP, and MDA were the lowest in the sham group, and the value of TAS was the highest in the sham group. With the exception of the TOS values between the sham and IRI + ABS groups (P = 0.231), all the other values reached statistical significance (P < 0.05). 

The IRI + ABS group was found to have lower levels of TOS, FOP, and MDA, and higher levels of TAS than the IRI group (P < 0.05). 

## 4. Discussion 

The most significant finding of this study was that ABS has hepatoprotective effects on experimental liver IRI in rats. According to both the histopathological and biochemical results of this study, the IRI + ABS group showed better results than the IRI group in terms of liver function and tissue samples after IRI was performed. Therefore, although ABS is known as a hemostatic agent, because of the antioxidant and anti-inflammatory components it could be an alternative treatment for hepatic IRI. 

Liver IRI is an important scenario which has been extensively studied in recent decades and remains a therapeutic challenge for clinicians. It is related to a number of clinical situations, such as hemorrhagic shock, resuscitation, trauma, liver transplantation, and liver resection. It results from a complicated pathological network with a combination of factors such as impairment of sinusoidal endothelial cells, inflammation, disturbance of microcirculation, activation of Kupffer cells, accumulation of leukocytes, activation of complement factors, oxidative stress, apoptosis, and necrosis [17,18]. Various pharmacological agents such as endothelin, adenosine and nitric oxide agonists, prostaglandins, antagonists, complement inhibiters, Kuppfer cell inactivators, neutrophil inactivators, antioxidants, heat shock protein, anti-apoptosis agents, metabolic agents, nuclear factor kappa** B inducers, and traditional Chinese medicine have been used for the treatment of hepatic IRI [19]. However, of these pharmacological agents, none has become a routine component in clinical practice and further studies are needed in this area. 

ABS is a medical plant extract containing Glycyrrhiza glabra, Urtica dioica, Alpinia officinarum, Thymus vulgaris, and Vitis vinifera [11]. ABS is made use of in clinical hemorrhages when bleeding cannot be controlled by conventional methods [20, 21]. ABS has been suggested as an alternative treatment method for different types of bleeding that are refractory to treatment with conventional procedures. ABS has also been shown to have efficient antiinfective, antineoplastic, and curative modulator properties. The current perspective for the use of ABS is to provide hemostasis and promote wound healing [22]. In addition to hemostatic effects, ABS also has antioxidant and antiinflammatory effects due to the integral components. For example, Thymus vulgaris shows an antioxidant effect with inhibition of lipid peroxidation and T reactive substance, thereby increasing antioxidant enzyme activity, and total antioxidant status. It also has an antiinflammatory effect due to inhibition of edema, leukocyte migration, and lipopolysaccharide-stimulated inflammation [23–26]. Glycyrrhiza glabra demonstrates antiinflammatory effects through inhibition of reactive oxygen species, decreasing lipopolysaccharide inflammatory mediators and providing protection against lipid peroxidation of liposomal membrane and antioxidant effect, as the low density lipoprotein is protected against oxidation [27]. Vitis vinifera increases antioxidant capacity by protecting against low density lipoprotein oxidation, and scavenging free radicals [28]. Alpinia officinarum exhibits antiinflammatory and antioxidant activity by binding into the active sides of cyclooxygenase 2 (COX 2) and behaving as COX 2 inhibitors [29]. Urtica dioica shows a greater antioxidant potential with reactive oxygen species scavenging capacity [30]. With the antioxidant and antiinflammatory effects of all these components, ABS can show a hepatoprotective effect on liver IRI, as was demonstrated in this study. 

Hasgul et al. showed the antioxidant effects of ABS on aspirin-related oxidative mucosal damage in a rat model of gastric injury [31], and the antiinflammatory effects of ABS were shown by Koçak et al. in experimental colitis [13]. However, to the best of our knowledge, this study is the first to have investigated the antioxidant and hepatoprotective effects of ABS in an experimental model of liver IRI. The results demonstrated that the IRI + ABS group had higher levels of albumin and lower levels of AST and ALT than the IRI group. Furthermore, the IRI + ABS group had lower levels of TOS, FOP and MDA, and higher levels of TAS than the IRI group. According to the histopathological results, the congestion scores were found to be statistically decreased in the IRI + ABS group compared to the IRI group. Although vacuolization and necrosis were seen to be higher within the IRI group compared to the IRI + ABS group, the difference was not statistically significant. This could have been the result of the limited sample sizes of the groups or the relatively shorter time for experimental reperfusion injury to occur than for necrosis and vacuolization. 

In conclusion, ABS has a strong hepatoprotective effect due to its antiinflammatory and antioxidant properties. Therefore, it may be suggested that ABS could be used as a potential therapeutic agent for IRI. 

## Disclaimers/Conflict of interest

Our study is a multidisciplinary study involving different departments such as general surgery, biochemistry and pathology. All authors contributed to the study. All authors confirmed that they have no conflicts of interest. All authors confirmed that they have no financal support for research.

## Contribution of authors

A.D. and K.K. conceived and planned the experiments. M.T, R.P, Y.Ü, S.H., and A.S developed the theory. A.D., K.K., C.E., F.K., M.Ş., İ.A.K., and E.P.E. carried out the experiment. A.D. wrote the manuscript with support from C.E., K.K., and A.S. M.T performed the analytic calculations and performed the numerical simulations. All authors interpreted the data, revised it critically and contributed to the final version of the manuscript.

## Informed consent

Ethics committee approval was obtained from Ankara Training and Research Hospital Animal Experiments Local Ethics Committee (Decision numbered 46 and dated 31.05.2018).
